# Identification of the glutamine synthetase (*GS*) gene family in four wheat species and functional analysis of *Ta4D.GSe* in *Arabidopsis thaliana*

**DOI:** 10.1007/s11103-022-01287-4

**Published:** 2022-06-18

**Authors:** Huayan Yin, Qian Sun, Xiaoqing Lu, Lufei Zhang, Yanchao Yuan, Cuiling Gong, Xiaoyan He, Wujun Ma, Ping Mu

**Affiliations:** 1 College of Agronomy, Qingdao Agricultural University, Qingdao, 266109 China; 2 Key Lab of Plant Biotechnology in Universities of Shandong Province, College of Life Sciences, Qingdao Agricultural University, Qingdao, China

**Keywords:** Wheat, Glutamine synthetase, Gene family, Abiotic stress tolerance

## Abstract

**Abstract:**

Drought stress can negatively impact crop yield and quality. Improving wheat yields under drought stress is a major objective of agronomic research. Glutamine synthetase (GS) is a key enzyme of nitrogen metabolism that is critical to plant growth and development in abiotic stress response. However, to date, no systemic characterization of the *GS* genes has yet been conducted in wheat and its close relatives. We identified a total of 15 *GS* genes in *Triticum aestivum* (2n = 6x = 42; AABBDD), as well as 9 *GS* genes in *Triticum dicoccoides* (2n = 4x = 28; AABB), 6 in *Aegilops tauschii* (2n = 2x = 14; DD), and 5 in *Triticum urartu* (2n = 2x = 14; AA). The 35 *GSs* were further clustered into five lineages according to the phylogenetic tree. Synteny analysis revealed that the three subgenomes in bread wheat retained extensive synteny between bread wheat and its three relative species. We identified three up-regulated *TaGSs* (*Ta4A.GSe*, *Ta4B.GSe*, and *Ta4D.GSe*) from transcriptome data after drought and salt stress. *Ta4D.GSe* was subsequently used for further functional studies, and its subcellular localization were determined in *Arabidopsis* protoplasts. Its overexpression in *Arabidopsis* enhanced drought tolerance by increasing the ability of scavenging of reactive oxygen species (ROS) and osmotic adjustment. We identified *GS* gene family in four wheat species and performed comparative analyses of their relationships, chromosome locations, conserved motif, gene structure, and synteny. The subcellular localization of Ta4D.GSe was detected and its drought tolerance function was demonstrated. Taken together, these findings provide insight into the potential functional roles of the *GS* genes in abiotic stress tolerance.

**Key message:**

This report clearly shows detailed characterization of GS gene family in four wheat species and demonstrates that *Ta4D.GSe* plays an important role in enhancing drought tolerance by improving the scavenging of ROS and osmotic adjustment ability in *Arabidopsis*.

**Supplementary Information:**

The online version contains supplementary material available at 10.1007/s11103-022-01287-4.

## Introduction

Abiotic and biotic stresses are major environmental threats that result in considerable losses in crop productivity worldwide. In response to various stress factors, plants commonly exhibit osmotic and oxidative stresses (Baillo et al. 2019). One of the common responses shown by plants to mitigate stresses is the synthesis and accumulation of organic solutes known as osmoprotectants, such as proline (Pro), glycine betaine, O-sulphate, choline, sugars and polyols (Iqbal et al. 2014). Pro is a highly soluble neutral compound and mainly studied in response to osmotic stress (Ma et al. 2019; Verbruggen et al. 2008). It can stabilize antioxidant system through osmotic adjustments and protecting the integrity of cell membranes, thereby diminishing the impacts of reactive oxygen species (ROS; Reddy et al. 2015). It also can directly neutralize ROS, and may scavenge ·OH through a reaction that converts this amino acid to γ-aminobutyric acid (Sharma and Dieta 2006; Hayat et al. 2012; Signorelli et al. 2014). Multiple studies have proven that the importance of elevated Pro level in several plants exposed to varied stresses (e.g., drought, salt, chilling, heat, metal/metalloid and UV-B radiations) (Iqbal et al. 2014; Szabados and Savoure 2010).

Two pathways for Pro biosynthesis have been proposed in plants: glutamate (Glu) and ornithine (Orn) pathways. In Glu pathway, the biosynthesis of Pro begins with the phosphorylation of Glu to form γ-glutamyl phosphate, which is reduced by the action of bifunctional enzyme Δ^1^-pyrroline-5-carboxylate synthetase (P5CS; EC2.7.2.11/1.2.1.41) to glutamic-5-semialdehyde (GSA), which is spontaneously cyclized into pyrroline-5-carboxylate (P5C). Finally, P5C is reduced to Pro by the enzymatic catalysis of Δ^1^-pyrroline-5-carboxylate reductase (P5CR; EC 1.5.1.2). In this pathway, the biosynthesis of Pro takes place in the cytosol and chloroplasts, and glutamate is mainly derived from the glutamine synthetase-glutamine oxoglutarate aminotransferase (GS-GOGAT) cycle. In Orn pathway, Orn is trans-aminated to GSA through the activity of ornithine δ-aminotransferase (δ-OAT; EC 2.6.1.13), and subsequently gets converted to Pro via P5C (Szabados and Savoure 2010). It has been proved that biosynthesis of Pro in *Arabidopsis* occurs exclusively via the Glu pathway (Funck et al. 2008). Therefore, the Orn pathway remains controversial. Under osmotic stress, the biosynthesis of Pro by Glu pathway through enhancing GS-GOGAT cycle is the dominant pathway (Rejeb et al. 2014).

In most plant species, GS exists in multiple enzyme forms with a single isoform in the chloroplast (GS2) and up to five isoforms in the cytosol (GS1) (Swarbreck et al. 2011). GS2 is mainly involved in assimilation of NH_4_^+^, which is originated from nitrate reduction and photorespiration (Pérez-Delgado et al. 2015). The function of GS1 is mainly involved in the transport of storage nitrogen during seed germination and the reuse of nitrogen during leaf senescence (Harrison et al. 2003). In addition, GS is involved in grain protein synthesis. Nigro et al. (2017) isolated and confirmed that GS2 and Fd-GOGAT were related to grain protein accumulation. Further detailed analysis of the GS2 promoter showed that NAC transcription factor was involved in regulating its expression. Habash et al. (2010) also showed that overexpression of *GS* could increase the biomass and yield of transgenic plants.

GS is important for osmotic stress tolerance in plants. Szabados and Savoure (2010) showed that Pro accumulated in plants mainly via the GS-GOGAT pathway under drought stress. The *GS2* mutant of *Lotus japonicus* showed lower Pro accumulation and rehydration ability than did the wild-type under drought stress (Díaz et al. 2010). In addition, overexpression of *GS1* and *GS2* in tobacco resulted in a higher accumulation of sucrose, Pro, and chlorophyll, and an enhanced ability to scavenge ROS, thus improving tolerance to drought induced stress (Yu et al. 2020).

Previous studies suggested that GS is one of the important physiological indicators for plants to adapt to drought stress. Results from experiments with drought-sensitive and drought-tolerant wheat genotypes showed that GS and RuBisCO (Ribulose-1,5-bisphosphate carboxylase/oxygenase, EC 4.1.1.39) could be used as physiological indicators to detect drought adaptation in wheat (Nagy et al. 2013). Singh and Ghosh (2013) showed that the expression of *OsGS2* and *OsGS1;1* may be related to the drought tolerance of Khitish (a drought-tolerant rice variety) under drought stress.

In addition, GS is involved in maintaining carbon and nitrogen balance in plants. Drought stress could limit the absorption of inorganic nitrogen in plants, inhibit the synthesis of carbohydrate and protein in leaves, and promote the degradation of protein and carbohydrate, thus break the balance of carbon and nitrogen metabolism in plants (Xu and Zhou 2006). Notably, GS can effectively utilize organic nitrogen and participate in metabolic processes such as photorespiration, reduction of ammonia, and reassimilation of circulating ammonia, thus improve drought tolerance of plants (Kusano et al. 2011).

In wheat, GS isoenzymes can be divided into four subfamilies on the basis of their sequence homology and cellular location. The first subfamily consists of GS2a, GS2b, and GS2c, which are nuclear-encoded (on chromosome 2) and chloroplast-localized proteins. The second subfamily consists of GS1a, GS1b, and GS1c, the third consists of GSr1 and GSr2, and the fourth GSe1 and GSe2. GS1, GSr and GSe are also nuclear-encoded (on chromosome 6, 4, 4, respectively) and cytoplasm-localized proteins (Nigro et al. 2017; Bernard et al. 2008; Habash et al. 2007). Bernard and Habash (2009) showed that GS isozymes have different functions in nitrogen metabolism of wheat. TaGS1 (GS1.1) and TaGSr (GS1.2) were mainly involved in the reuse of nitrogen in senescent leaves. In summary, there are many studies on the relationship between GS activity and nitrogen use efficiency and yield, and studies on the response of GS to abiotic stress such as drought, salt and extreme temperature are increasing gradually. The molecular mechanism of GS resistance to abiotic stress has attracted more and more attention in recent years.

Here, we aim to carry out a comprehensive study on the molecular characterization, phylogenetic relationship, and expression profiling of wheat *GS* gene family from the four wheat species, *Triticum aestivum* (*Ta*), *Triticum dicoccoides* (*Td*), *Aegilops tauschii* (*Aet*), and *Triticum urartu* (*Tu*). In addition, we infer that *TaGSs* respond to drought and salt stress through transcriptome data. The overexpression of *Ta4D.GSe* in *Arabidopsis* was used to confirm the effectiveness on drought tolerance of *Ta4D.GSe*.

## Materials and methods

### Data search and sequence retrieval

The genome files and annotation gff3 files of *T. aestivum* L. (Chinese spring), *Triticum turgidum* ssp. *dicoccoides* (Zavitan), *A. tauschii* Coss. (AL8/78) and *T. urartu.* (G1812) were downloaded from the EnsemblPlants (http://plants.ensembl.org) and MBKBASE (http://www.mbkbase.org). The published GS protein sequences were obtained from NCBI database (https://www.ncbi.nlm.nih.gov/protein), including *Oryza sativa* (*Os*), *Zea mays* (*Zm*), *Hordeum vulgare* (*Hv*), *Arabidopsis thaliana* (*At*), *T*), and six microbial species or genera *Mucor ambiguus* (*Ma*), *Isosphaera pallida* (*Ip*), *Leptolyngbya* (*Le*), *Phaeodactylibacter* (*Ph*), *Caldithrix abyssi* (*Ca*) and *Phaeodactylum tricornutum* (*Pt*). All of the 45 published GS proteins were used as query sequence to scan the whole genome protein sequences of bread wheat and its relative species with BLAST algorithm for Proteins (BLASP) search (e-value < 1e−5).

### Genome-wide identification and characterization of *GS* genes

All candidate GS protein sequences were identified using the NCBI Conserved Domain Database (CDD, https://www.ncbi.nlm.nih.gov/cdd) with the automatic model and default parameters (threshold = 0.01, maximum hits = 500) and confirmed in InterPro (http://www.ebi.ac.uk/interpro/). The conserved protein domains in confirmed GS proteins were filtered from the CDD results.

The localization on chromosomes of all *GS* genes were analyzed by TBtools (Chen et al. 2020) using the annotation gff3 files. The number of amino acids, molecular weight, isoelectric point, and grand average of hydropathicity of GS proteins were analyzed by ProtParam (Gasteiger et al. 2005) (https://web.expasy.org/protparam/). The subcellular localizations were obtained from the web-server CELLO v2.5 (Yu et al. 2006) (http://cello.life.nctu.edu.tw/).

### Synteny analysis and phylogenetic construction of *GS* genes

For the synteny analysis, the whole genome protein sequences of four wheat species were first pairwise compared by BLAST, then the calculation of the collinearity examination of paralogous genes were performed with MCScanX (http://chibba.pgml.uga.edu/duplication/) in TBtools. Finally, synteny visualization was conducted by TBtools.

The phylogenetic tree was constructed using the maximum likelihood method with MEGA X software (Kumar et al. 2018). At first, the GS proteins were aligned by Clustal W with default parameters. Then, a maximum likelihood phylogenetic tree was constructed, using the Poisson model, with 1000 bootstrap replicates. We colored the tree by web-server ITOL (https://itol.embl.de/) afterwards.

### Gene structure and conserved motif analysis

The exon–intron structures of *GS* genes were constructed by Gene Structure Display Server 2.0 (GSDS, http://gsds.cbi.pku.edu.cn/). MEME v5.1.0 (http://meme-suite.org/tools/meme) was employed to analyze the conserved motifs of *GS* genes. We used the following parameters: distribution of motif occurrences, 0 or 1 occurrence per sequence; motif width 6 to 50 bp; and maximum number of motifs, 24. Finally, the analyzed results were visualized by TBtools.

### Expression analysis of *TaGS* genes

According to gene expression profiles of Qingmai6 under 15% (w/v) polyethylene glycol (PEG) 6000 and 200 mmol·L^−1^ NaCl treatment (unpublished data), the differential expression of *TaGS* genes before and after treatment were obtained and analyzed. Then the expression characteristics were normalized and displayed as a heatmap. To detect the candidates that show the highest expression yields, we further checked the expressions of *Ta4A.GSe*, *Ta4B.GSe*, and *Ta4D.GSe* in Qingmai6 leaves with RT-qPCR after PEG-6000 (20% w/v, 2 h) treatment. The tissue specific expression of *Ta4D.GSe* was also detected by quantitative reverse-transcription PCR (RT-qPCR) at Qingmai6 seedling stage and maturation stage. According to the expression characteristics of *Ta4D.GSe*, it was induced by abiotic stress (PEG and salt) in Qingmai6. The function of *Ta4D.GSe* in drought tolerance was verified by comparing its levels of expression in three drought-tolerant wheat varieties (Qingmai6, Lumai21, and Shanrong3) (Zhang et al. 2011; Peng et al. 2009) and one drought-sensitive (Chinese Spring) (Hao et al. 2015) when exposed to drought stress conditions. The wheat seedlings were grown in nutrient solution, a continuous 25℃ temperature, a photoperiod of 12/12 h, and 50% relative humidity were used in a growth chamber. Drought stress treatment was carried out by submerging wheat seedling roots in nutrient solution of 20% (w/v) PEG-6000 at three-leaf stage. Leaves of the seedlings were sampled at different time points (0, 12, 24, 48, and 72 h) after treatment and RT-qPCR was used to detect *Ta4D.GSe* expression in the different wheat varieties.

Total RNA was extracted with the Total RNA Extraction Kit (Solarbio), and then one microgram of total RNA was reverse transcribed using PrimeScript™ RT reagent Kit (Perfect Real Time, Takara). In the RT-qPCR, β-actin gene was used as internal reference gene. For RT-qPCR, SYBR Green system (Roche) was used. Each experiment was repeated at least three times, with each reaction performed in triplicates, and the relative expression values were analyzed with the 2^−ΔΔCq^ method. All tests were two-sided and p < 0.05 was considered statistically significant. All the gene-specific primers used for amplifications or vector constructions were listed in Table S1.

### Subcellular localization and overexpression of *Ta4D.GSe* in *Arabidopsis thaliana*

Following the procedures of Wu et al. (2009) and Chen et al. (2006), we cloned and fused the non-terminator coding sequences of *Ta4D.GSe* to a green fluorescent protein (GFP) into vector Super1300 and transfected 10 μg plasmid DNA to 2 × 10^4^ protoplasts by transient expression in 3-week-old *Arabidopsis* mesophyll protoplast method. GFP fluorescence was observed with a confocal laser scanning confocal microscope TCSsp5II (Agilent). In addition, the full coding sequence of the *Ta4D.GSe* was cloned into vector pBI121 with CaMV 35S promoter for gene overexpression. The recombinant plasmid and empty vector were introduced into the *Agrobacterium tumefaciens* strain EHA105, which were then infiltrated into *At* wild-type (Col-0) plants for transformation (Clough and Bent 1998). The T3 generation of transgenic *Ta4D.GSe* overexpression lines (Ta4D.GSe-OE) were used for further phenotypic analysis. The empty vector transgenic lines were used as mock, to serve as control, and will be referred to as WT.

### Drought tolerance assessment

Surface sterilized seeds of *At* and transgenic lines were planted on Murashige and Skoog medium (MS). All of those were vernalized at 4℃ in dark for 3 days, and then grown under sterile conditions with 22℃, at a 16 h light/8 h dark cycle. For relative germination rate detection, the MS medium was added in different concentrations (50 mM, 100 mM, 150 mM) of mannitol. After 5 days, the relative germination rates were counted. For the detection of survival rate, 10 days after germination, seedling were grown in chamber with an equal weight of dry commercial soil (PINDSTRUP): vermiculite (1:3, v:v) at 22℃ under 16 h light/8 h dark cycle and 70% relative air humidity. Two-week-old seedlings were exposed to drought by discontinuing irrigation for 7 days and then re-watered with 100 mL water, which was the drought–rewatering treatment cycle. After 3 drought treatment cycles, the survival rate was determined based on the method of Li et al. (2020). After ten days of drought treatment, the enzyme reagent boxes (Solarbio) were used to detect the activities of GS, superoxide dismutase (SOD, EC 1.15.1.1), peroxidase (POD, EC 1.11.1.7) and catalase (CAT, EC 1.11.1.6). In addition, soluble sugars content was determined by the anthrone assay (Wang et al. 2013), and ninhydrin spectrophotometric assay (Bates et al. 1973) was used to detect the Pro level. Each experiment described above was repeated at least three independent times. All tests were two-sided and p < 0.05 was considered statistically significant.

## Results

### Genome-wide identification and characterization of *GS* genes in bread wheat and its relatives

Blast searches were performed by querying *Os*, *Zm*, *Hv* and *At* GS protein sequences from different wheat genomes, and 6, 15, 9 and 5 candidate *GS* genes were found in *Aet*, *Ta*, *Td* and *Tu*, respectively. The characteristics of 35 GS proteins in four wheat species are shown in Table 1. The coding amino acid length of four wheat species was between 60 and 884, and the molecular weights ranged from 6.15 to 98.33 kDa. Two proteins TRIDC4AG008800.5 (7.05) and TRIDC6BG052800.5 (7.67) showed isoelectric point above 7, indicating that these proteins were alkalescent, while all others showed isoelectric point below 7 indicating that they all were acidulous. In addition, all GS proteins in four wheat species had a negative grand average of hydropathicity (GRAVY) score, indicating that they were hydrophilic in nature.

**Table 1:** Characteristics of 35 GS proteins in four wheat species

Species	Clade	Sequence IDs	Chromosome	Number of amino acid	Molecular weight (kDa)	Isoelectric point	Grand average of hydropathicity (GRAVY)	Most-likely-location
*Ae. tauschii*	I	AET1Gv20368100.2	1D	842	93.25	5.68	− 0.108	Cytoplasmic
		AET6Gv20169200.31	6D	843	93.29	6.09	− 0.115	Cytoplasmic
	III	AET4Gv20601300.11	4D	354	38.8	5.71	− 0.349	Cytoplasmic/periplasmic/extracellular
		AET6Gv20470300.3	6D	60	6.15	4.9	− 0.002	Cytoplasmic/periplasmic
	IV	AET4Gv20094400.1	4D	440	48.25	6.05	− 0.412	Cytoplasmic
	V	AET6Gv20743700.1	6D	371	40.76	5.69	− 0.383	Periplasmic
*T. aestivum*	I	TraesCS1A02G143000.1	1A	841	93.27	5.85	− 0.135	Cytoplasmic
		TraesCS1B02G158600.1	1B	842	93.32	5.85	− 0.112	Cytoplasmic
		TraesCS1D02G141800.1	1D	842	93.25	5.68	− 0.108	Cytoplasmic
		TraesCS6D02G065600.1	6D	815	90.28	6.22	− 0.111	Cytoplasmic
	II	TraesCS2A02G500400.1	2A	427	46.7	5.75	− 0.338	Periplasmic/cytoplasmic
		TraesCS2B02G528300.1	2B	423	46.08	5.89	− 0.32	Periplasmic/cytoplasmic
		TraesCS2D02G500600.1	2D	427	46.7	5.75	− 0.338	Periplasmic/cytoplasmic
	III	TraesCS4A02G063800.1	4A	354	38.69	5.45	− 0.356	Cytoplasmic
		TraesCS4B02G240900.1	4B	354	38.73	5.35	− 0.366	Cytoplasmic
		TraesCS4D02G240700.1	4D	354	38.66	5.34	− 0.355	Cytoplasmic
	IV	TraesCS4A02G266900.1	4A	362	39.61	5.3	− 0.438	Cytoplasmic/extracellular/periplasmic
		TraesCS4B02G047400.1	4B	362	39.47	5.66	− 0.416	Cytoplasmic
		TraesCS4D02G047400.1	4D	362	39.48	5.53	− 0.423	Cytoplasmic
	V	TraesCS6A02G298100.2	6A	356	39.2	5.41	− 0.387	Periplasmic/cytoplasmic
		TraesCS6B02G327500.1	6B	356	39.21	5.41	− 0.394	Periplasmic/cytoplasmic
*T. dicoccoides*	I	TRIDC1AG021640.1	1A	884	98.33	6.56	− 0.201	Cytoplasmic
		TRIDC1BG025770.5	1B	611	67.98	6.16	− 0.156	Cytoplasmic
	II	TRIDC2BG076090.1	2B	427	46.69	5.75	− 0.338	Periplasmic/cytoplasmic
	III	TRIDC4AG008800.5	4A	243	26.41	7.05	− 0.391	Periplasmic/cytoplasmic
		TRIDC4BG042280.3	4B	161	17.68	6.2	− 0.47	Cytoplasmic
	IV	TRIDC4AG041670.2	4A	395	43.48	5.62	− 0.474	Cytoplasmic
		TRIDC4BG007380.7	4B	418	45.69	6.59	− 0.432	Periplasmic/cytoplasmic/extracellular
	V	TRIDC6AG045200.3	6A	253	28.11	6.78	− 0.419	Cytoplasmic/periplasmic
		TRIDC6BG052800.5	6B	420	46.07	7.67	− 0.38	Periplasmic
*T. urartu*	I	TuG1812G0195861400.01.T02	1A	845	93.87	5.96	− 0.131	Cytoplasmic
	II	TuG1812G0205340600.01.T06	2A	780	87.78	5.36	− 0.343	Cytoplasmic
	III	TuG1812G0410057100.01.T02	4A	354	38.69	5.45	− 0.356	Cytoplasmic
	IV	TuG1812S0003369600.01.T01	Un	362	39.62	5.31	− 0.443	Cytoplasmic/periplasmic
	V	TuG1812G0615480200.01.T03	6A	398	44.01	6.16	− 0.302	Periplasmic

**Fig. 1:**
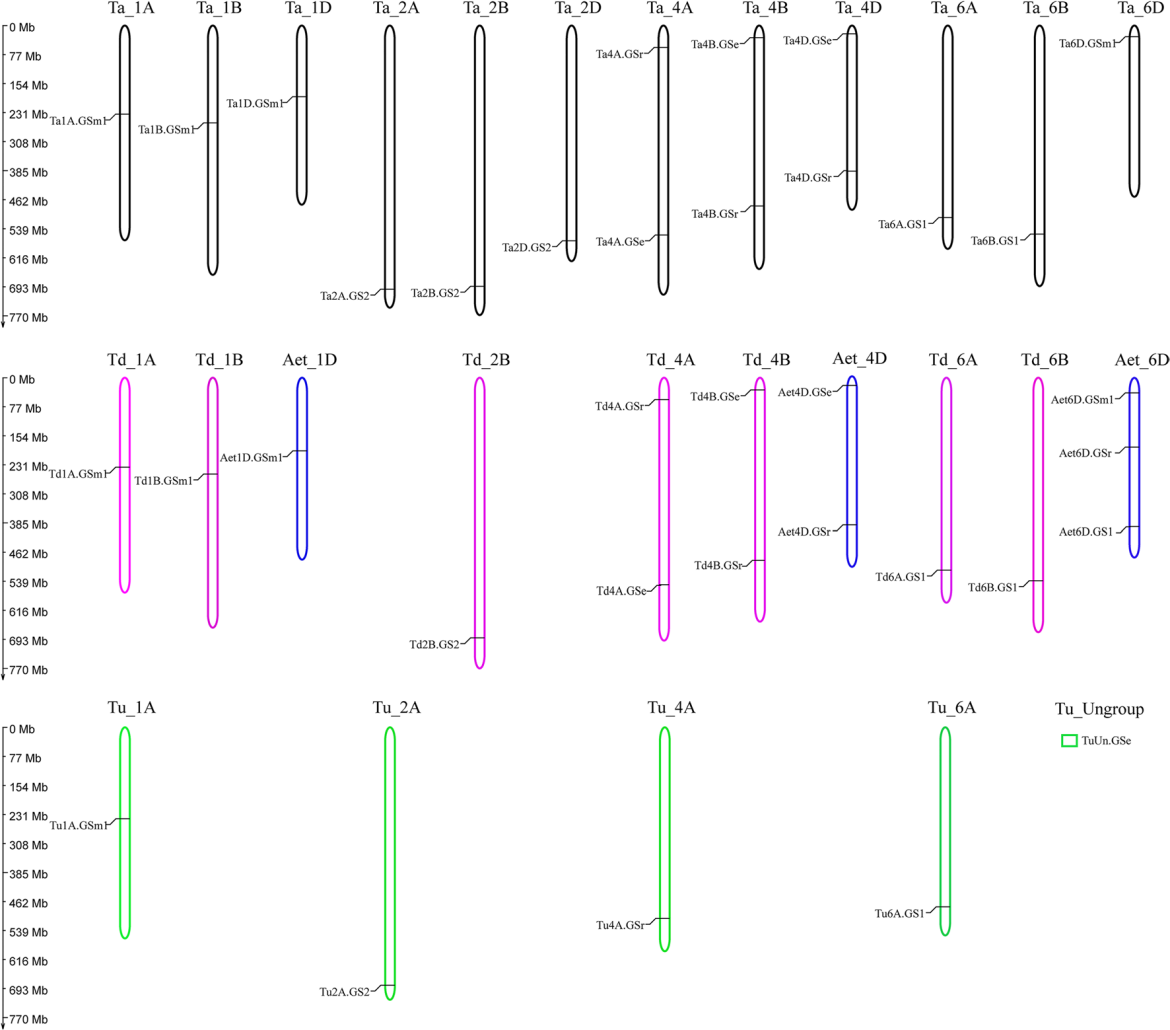
Chromosome distribution of *GS* genes of four wheat species. The vertical scale on the left showed the physical size of chromosomes and black lines indicated the position of genes. The gene names correspond to those in Table S1

According to the genomic location of each member of the *GS* gene family, a chromosome location map was constructed to illustrate the distribution of the *GS* genes by TBtools (Fig. 1; Table S1). The *GS* genes existed on the chromosomes from the groups 1, 2, 4, and 6 in *Ta*, *Td* and *Tu*, with number of *GS* genes in each chromosome ranging from one to two, and majority were detected at the distal end of the chromosomes. However, *GS* genes were located on 1D, 4D, and 6D in *Aet*. No *GS* gene was located on the chromosomes from the groups 3, 5, and 7 of all four wheat species. The exception here is *TuUnGSe* was located on chromosome *TuUn* in *Tu* because of the incomplete genome sequence. The chromosome 6D of *Aet* had 3 genes, the largest number of *GS* genes found in a single chromosome. The numbers of *GS* genes distributed in the subgenome showed little difference. The *GS* numbers in A, B and D subgenome of *Ta* were all 5, while a total of 4 and 5 *GS* genes were located on subgenomes A and B of *Td*, respectively. What is more, the *GS* numbers in A subgenome of *Tu* and D subgenome of *Aet* were 5 and 6.

### Synteny analysis of *GS* genes among four wheat species

**Fig. 2:**
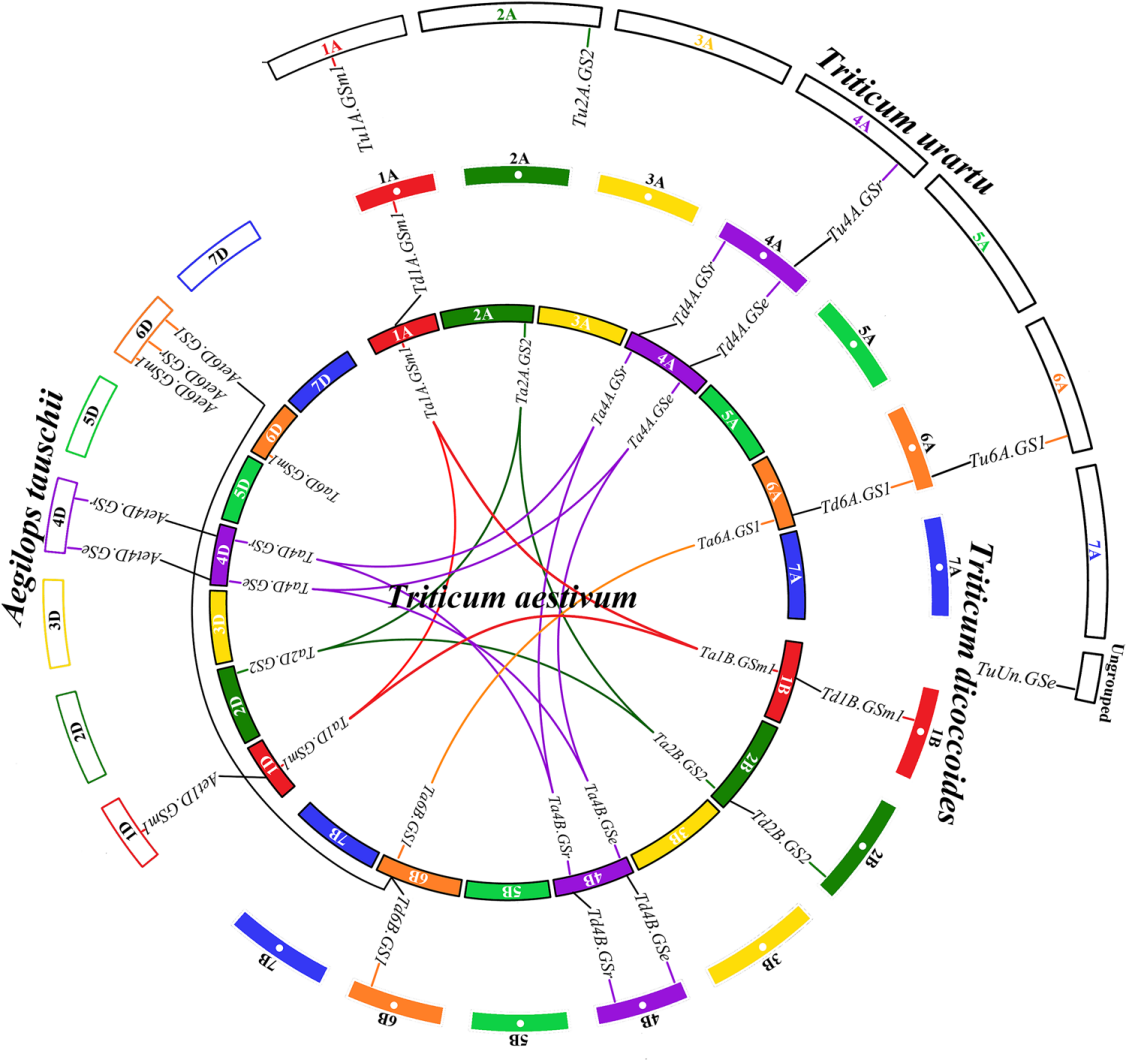
Synteny analyses of *GS* genes between *Triticum aestivum*, *Triticum dicoccoides*, *Aegilops tauschii* and *Triticum urartu*

Among all the 15 *GS* genes of *Ta*, 4 *TaGSs* had intergenomic homologous genes in *Aet*, 9 homologous genes in *Td* and 4 homologous genes in *Tu*, respectively (Fig. 2). The synteny analysis illustrated that four *Aet-GSs* could be mapped to bread wheat D subgenomes on the same chromosomes with one on 1D, two on 4D, except for *Aet6D.GS1*/*Ta6B.GS1* homologous gene pairs (*Aet6D.GS1* on 6D, *Ta6B.GS1* on 6B). Moreover, nine *Ta*/*Td.GSs* homologous gene pairs were located on the same chromosomes, with one on 1A, two on 4A, one on 6A, one on 1B, one on 2B, two on 4B and one on 6B. Furthermore, only two homologous gene pairs were found between *Tu* and *Td*, with one on 4A and one on 6A.

### Phylogenetic analysis of *GS* genes

**Fig. 3:**
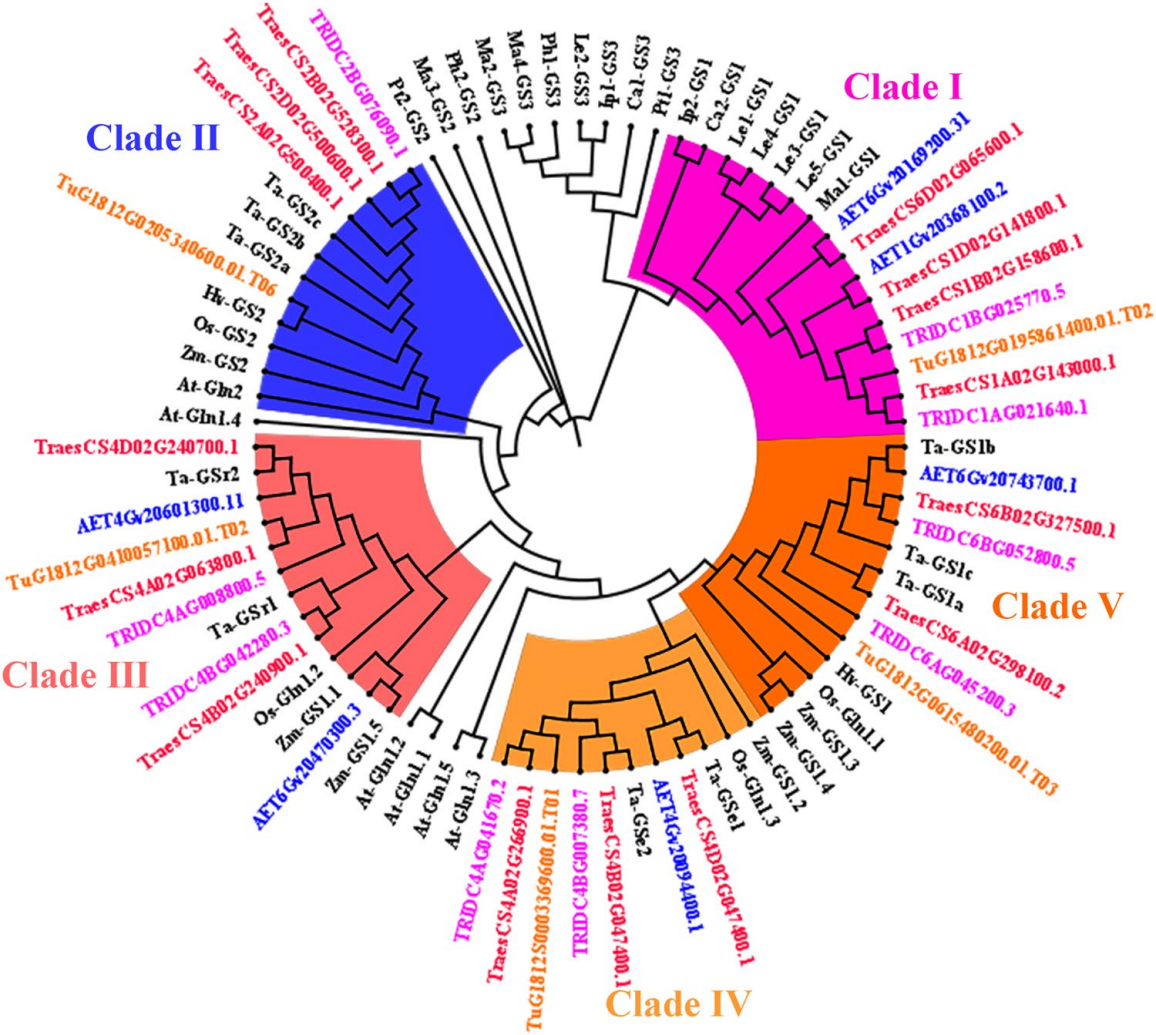
Phylogenetic tree of 80 GSs homolog proteins from different species. The GS proteins from 6 microbial species *Mucor ambiguus* (*Ma*), *Isosphaera pallida* (*Ip*), *Leptolyngbya* (*Le*), *Phaeodactylibacter* (*Ph*), *Caldithrix abyssi* (*Ca*) and *Phaeodactylum tricornutum* (*Pt*), and 4 model species *Oryza sativa* (*Os*), *Zea mays* (*Zm*), *Hordeum vulgare* (*Hv*), and *Arabidopsis thaliana* (*At*), and 10 *Triticum aestivum* (*Ta*). Five major clades were distinguished with colors

To study the phylogeny and subgroups of the *GS* family, an unrooted phylogenetic tree was constructed by using the 35 putative GS protein sequences from bread wheat and its relative species, and 4 *OsGSs*, 6 *ZmGSs*, 2 *HvGSs*, 5 *AtGSs*, 10 *TaGSs* and 17 *GSs* obtained from 6 micro-organisms in the literature (Table S2). Based on sequence analysis, phylogenetic studies and mapping data, Bernard et al. (2008) classified 10 *TaGSs* into four subfamilies: *GS2*, *GS1*, *GS*r, and *GSe*. However, in the phylogenetic tree, the 35 *GSs* were divided into five major clades, including 9 novel genotypes belonging to clade I, which show a high identity level to the *GS* genes in micro-organisms (Fig. 3). Based on the sequence of micro-organisms, clade I was separated from the *GS* family for the first time as a separate subfamily, including 4 *TaGSs* (*TraesCS1A02G143000.1*, *TraesCS1B02G158600.1*, *TraesCS1D02G141800.1* and *TraesCS6D02G065600.1*), 2 *TdGSs* (*TRIDC1AG021640.1* and *TRIDC1BG025770.5*), 2 *AetGSs* (*AET1Gv20368100.2* and *AET6Gv20169200.31*), and 1 *TuGSs* (*TuG1812G0195861400.01.T02*). Therefore, the *GS* genes in clade I were expressed as *GSm1* (Table S1). According to the published sequences of *TaGSs*, *OsGSs*, *ZmGSs*, *HvGSs*, and *AtGSs*, clade II belongs to the *GS2* subfamily encoding nuclear gene for chloroplastic *GS2* isoenzyme. Moreover, the other three clades (III, IV, and V) encode cytosolic *GS1* isoenzymes (*GSr, GSe*, and *GS1*).

### Sequence and structural analyses of *GS* genes and proteins

**Fig. 4:**
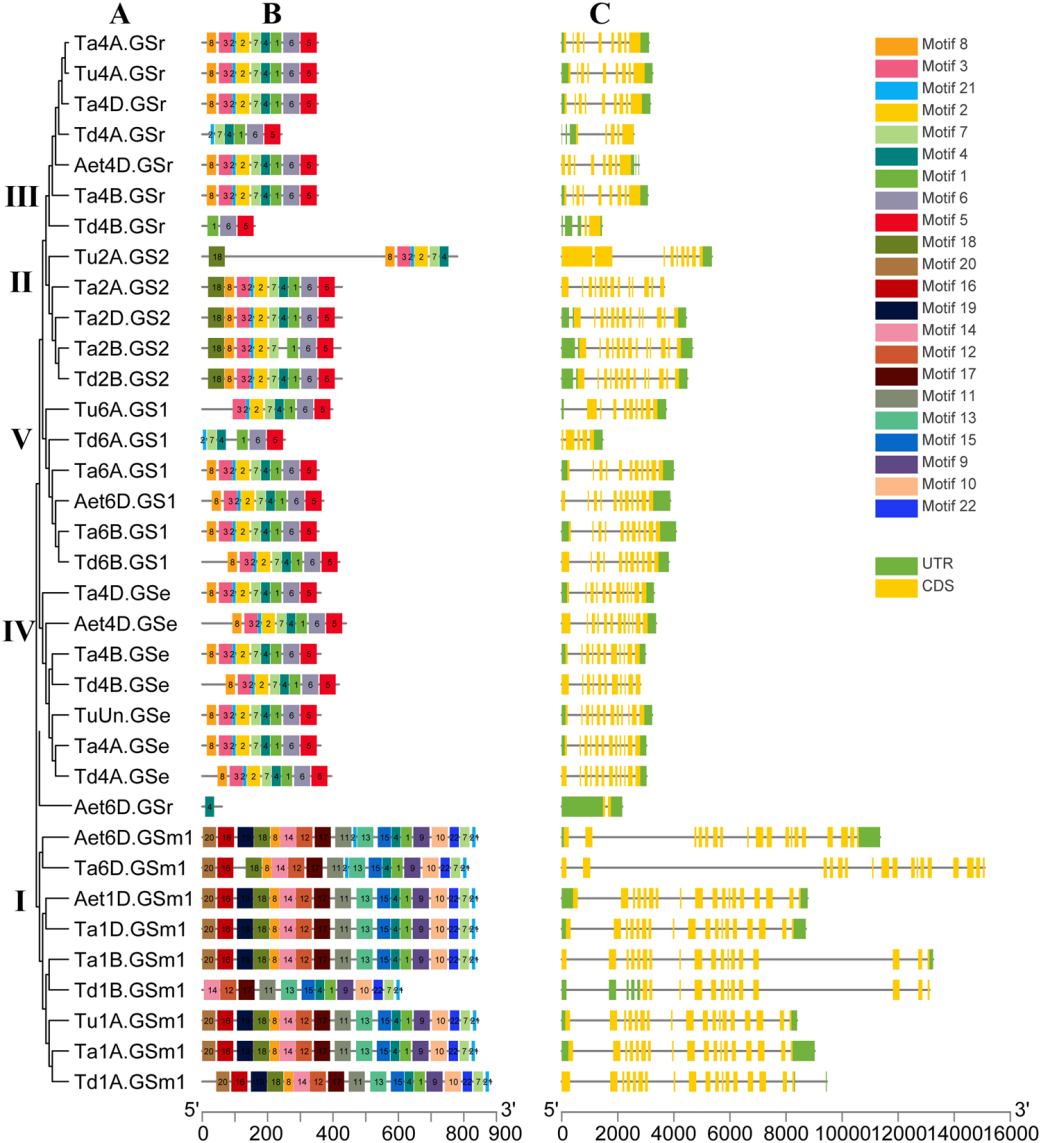
Phylogenetic, conserved motif and gene structure analyses of *GS* genes. **A** Phylogenetic analysis, **B** conserved motif, and **C** gene structure. *UTR* untranslated region, *CDS* coding sequence

The gene structure of *GSs* genes was analyzed according to the gene annotation gff3 files. As illustrated in Fig. 4 and Table S3, 22 conserved motifs, with 11 to 50 amino acids, of *GS* genes were identified through the MEME v5.1.0, and most of motifs displayed similar patterns within the *GS* genes in four wheat species. For example, motif 8, 3, 21, 2, 7, 4, 1, 6, 5 were conserved in 19 *GSs*, and motif 21, 7, 4, 1, 6, 5 were retained as part of the combination in two *GSs*, *Td4A.GSr* and *Td6A.GS1*. Compared with *GS1*, *GSr* and *GSe* subfamily, motif 18 was specific to the *GS2* branch. The number and type of conserved motifs were consistent in clade I, however, they were different from those in clade II to clade V. These results indicated that the *GSs* in different subfamilies had different conserved motif distributions, which might suggest a conserved function of different subfamilies. Gene structure analysis showed that a majority of the *GS* genes had more than one exon. In the same subfamily, the gene structure and conserved motif distribution were similar, indicating that the phylogenetic tree constructed in this study is accurate.

### RNA-seq expression profile of *TaGS* genes in abiotic stress

**Fig. 5:**
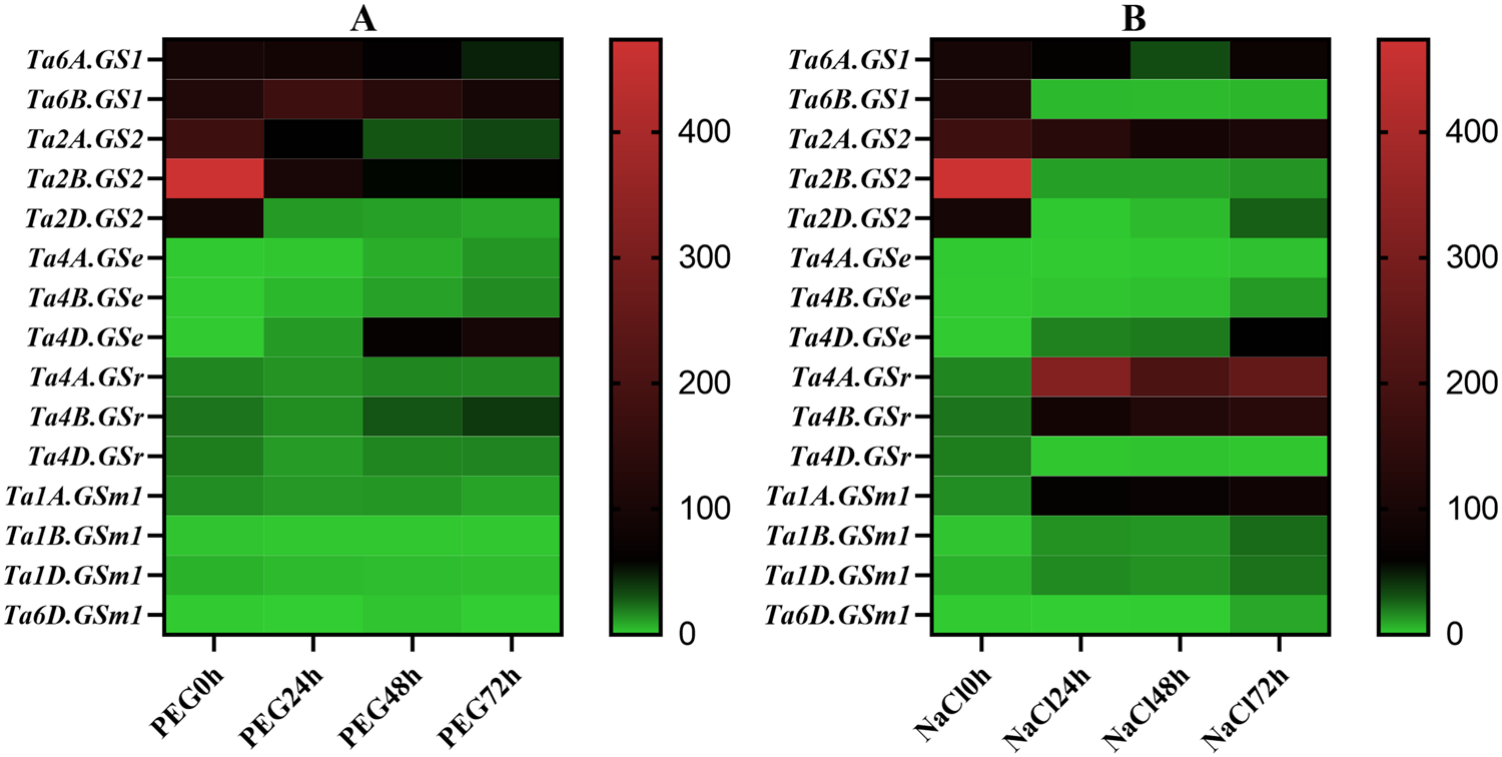
Heat map of the expression profiling of *TaGS* genes at different time under abiotic stress Green and red denote lower and higher expression levels, respectively. The lables 0 h, 24 h, 48 h, and 72 h indicate the time that passed after the PEG (**A**) and NaCl (**B**) treatment. Transcriptome expression of reads per kilobase per million mapped reads (rpkm) is the RNA-Seq expression unit

**Fig. 6:**
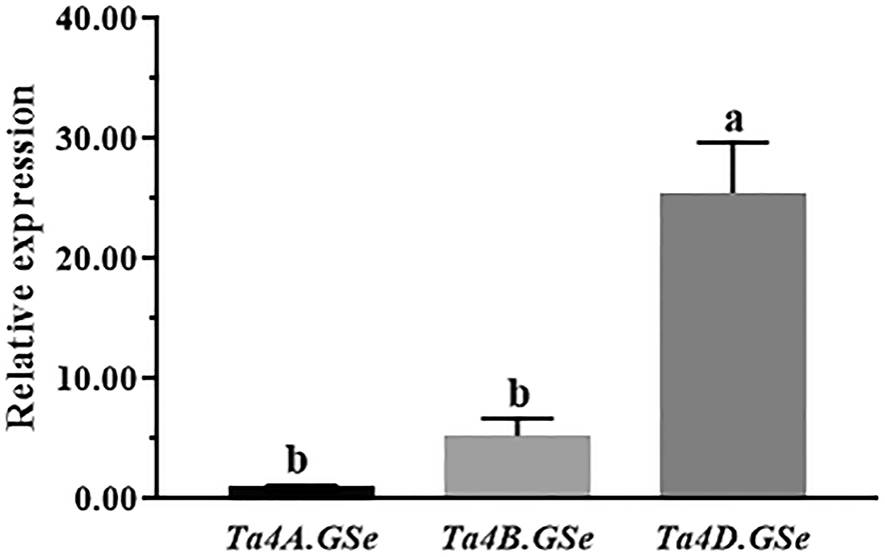
Expression levels of *Ta4A.GSe*, *Ta4B.GSe*, and *Ta4D.GSe* in leaves under drought stress. The relative expression levels were calculated by setting the expression value of *Ta4A.GSe* as 1. The relative expression values were calculated through the 2^−ΔΔCq^ approach

To identify the potential functions of *TaGS* genes in response to abiotic stress, the expression data under PEG and NaCl treatment were obtained from Qingmai6 RNA-Seq data, respectively. The expression profiles of *TaGSs* in leaf (0 h, 24 h, 48 h, 72 h) under abiotic stress were normalized to log_2_^FPKM^ and performed with heatmap (Fig. 5). Under both PEG and NaCl treatment, the expression of *Ta4A.GSe*, *Ta4B.GSe*, and *Ta4D.GSe* from subfamily *GSe* were significantly up-regulated, and *Ta4D.GSe* was the most strongly up-regulated gene. The results of RT-qPCR in Qingmai6 under PEG treatment were consistent with the RNA-seq data shown in the heatmap (Fig. 6). The expression levels of *Ta4D.GSe* in Qingmai6 different tissues at seedling stage and maturation stage were compared. The highest expression levels were found in root and spikelet, respectively (Fig. S1), indicating that *Ta4D.GSe* function mainly in those tissues.

**Fig. 7:**
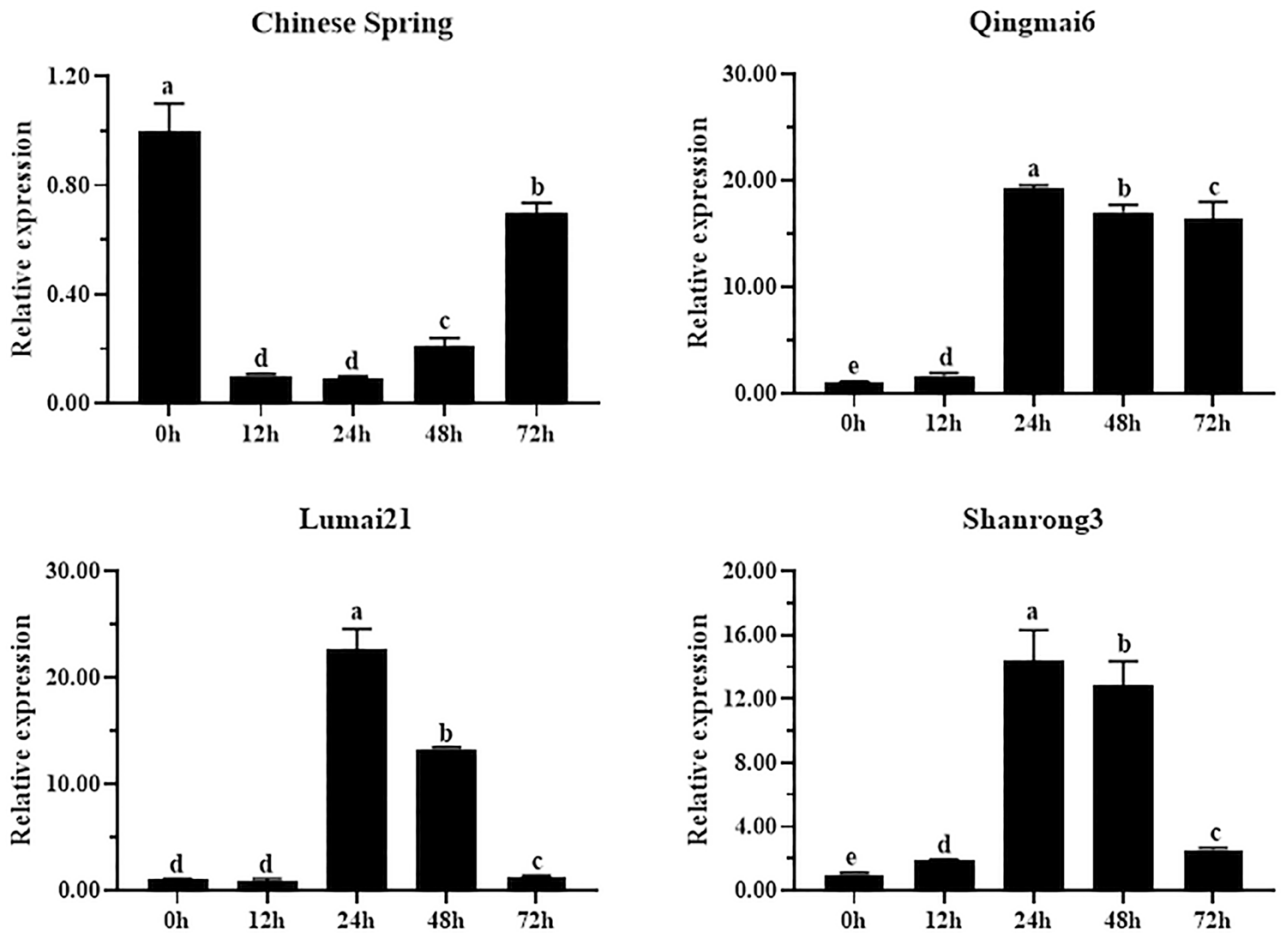
The expression pattern of *Ta4D.GSe* in different wheat varieties under PEG treatment. Drought-tolerant wheat varieties: Qingmai6, Lumai21, and Shanrong3. Drought-sensitive variety: Chinese Spring. The relative expression levels in each variety were calculated by setting the expression value at 0 h as 1. The relative expression values were calculated through the 2^−ΔΔCq^ approach

To confirm that the expression pattern of *Ta4D.GSe* was associated with drought tolerance, RT-qPCR were used to detect it in drought-tolerant wheat varieties (Qingmai6, Lumai21, Shanrong3) and drought-sensitive variety (Chinese Spring) under PEG treatment. As shown in Fig. 7, *Ta4D.GSe* from drought-tolerant wheat varieties were more sensitive, being significantly up-regulated under PEG treatment. Thus, based on the analysis above, *Ta4D.GSe* was selected as representative gene for further functional investigation.

### Subcellular localization and ectopic overexpression analysis of *Ta4D.GSe* in *Arabidopsis*

**Fig. 8:**
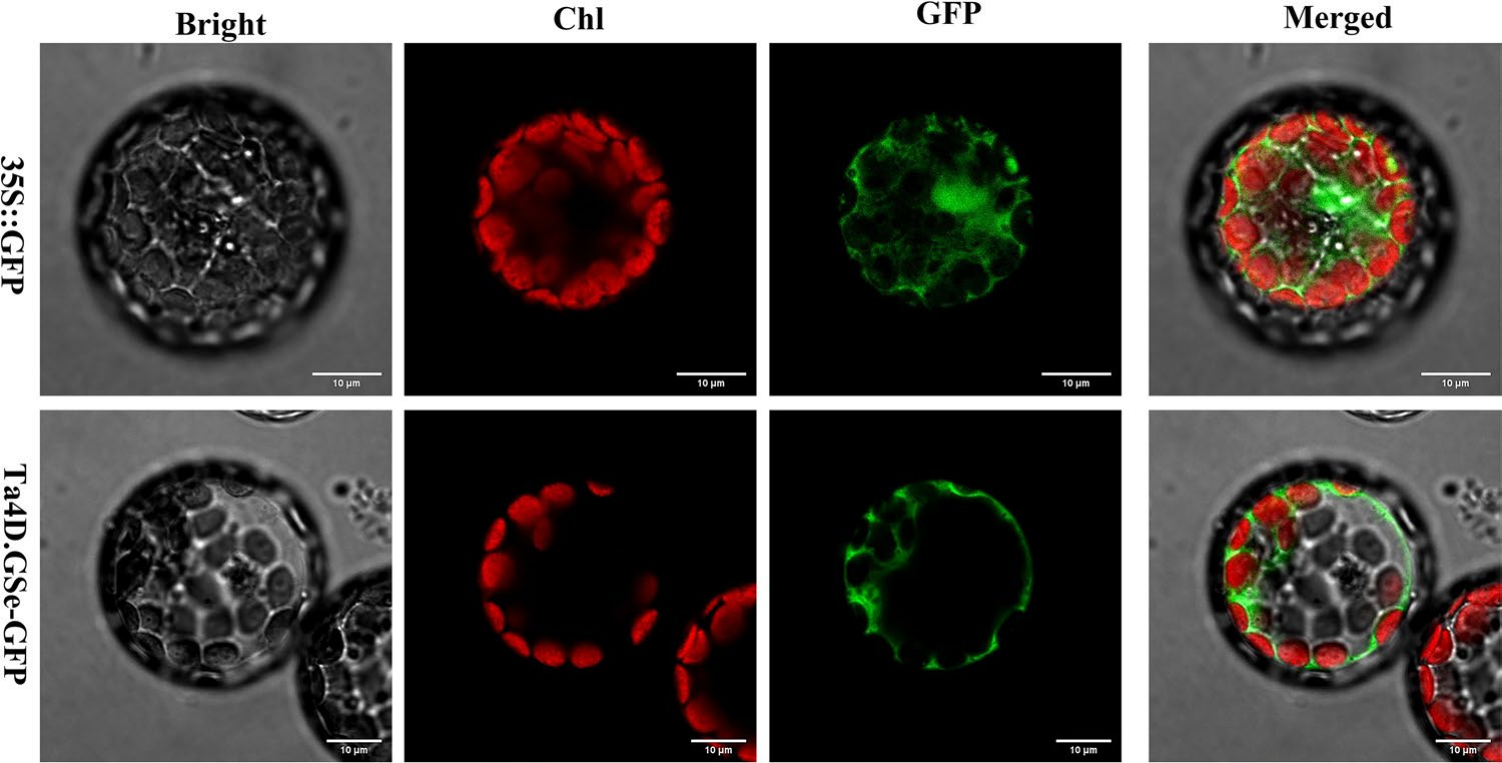
The subcellular localization of Ta4D.GSe. *Scale bar* 10 μm

**Fig. 9:**
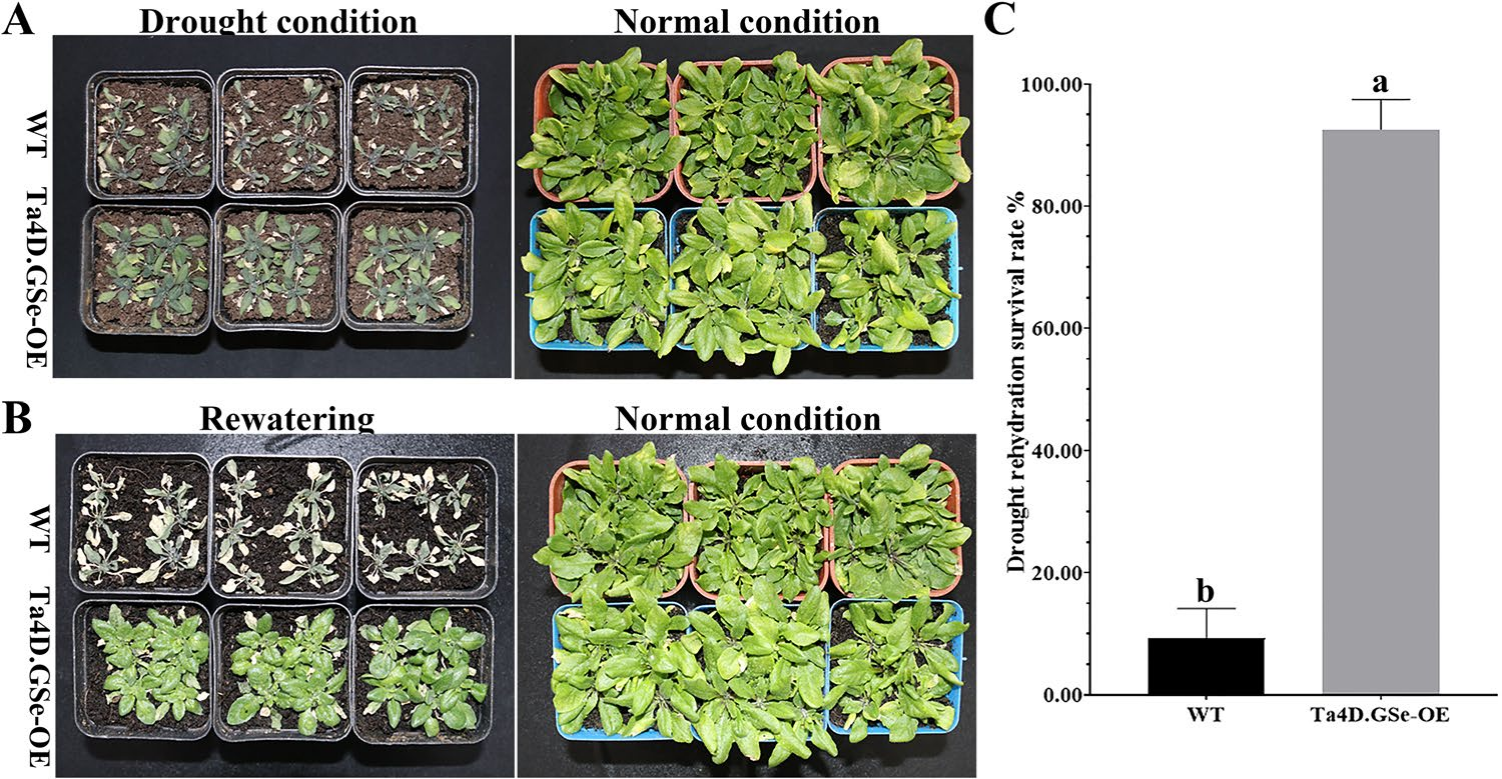
Overexpression of *Ta4D.GSe* enhances drought resistance in *Arabidopsis*. WT, empty vector lines. **A** Phenotype of wild-type and homozygous transgenic lines grown under normal and drought stress conditions. **B** Phenotype of wild-type and homozygous transgenic lines under normal and 5 days after rewatering conditions. **C** The survival rate of wild-type and homozygous transgenic lines (Ta4D.GSe-OE) at 5 days after rewatering conditions

The subcellular localization analysis showed that Ta4D.GSe was localized at the cytoplasm (Fig. 8), and the result was consistent with the subcellular localization prediction by CELLO v2.5 (Table 1). Compared to those of the WT, no significant difference were observed in biomass production under normal conditions, however, the germination rate and tolerance of seedlings under mannitol treatment and in response to repeated drought treatments were significantly improved (Fig. S2; Fig. 9), suggesting that the function of *Ta4D.GSe* is more evident following drought stress.

The activities of SOD, POD, and CAT of Ta4D.GSe-OE were significantly higher than that of WT before drought stress (Fig. S3). After 10 days of drought treatment, the enzymatic activities were significantly increased in both the Ta4D.Gse-OE and WT lines. The activities in SOD, POD, and CAT of Ta4D.GSe-OE were increased by 2.3, 1.2, and 1.8-fold, respectively, and in the WT, these enzyme’s activities were increased by 1.6, 1.1, and 1.6-fold, respectively, when compared with those of the untreated plants (Fig. S3b–d). The result indicated that *Ta4D.GSe* plays a significant role in conferring drought tolerance by improving the scavenging of ROS in *Arabidopsis*. In addition to the results above, soluble sugars and free-Pro accumulated in WT and Ta4D.GSe-OE and the accumulation in Ta4D.GSe-OE were much more significant than that in WT, after 10 days of drought treatment. The content of soluble sugars and free-Pro in Ta4D.GSe-OE were increased by 4.1 and 24.8-fold, respectively, and in WT were increased by 2.8 and 21.4-fold, respectively (Fig. S3e, f). The result showed that *Ta4D.GSe* could improve the osmotic adjustment ability in *Arabidopsis* through accumulate osmoregulating substances.

## Discussion

Whole genome-wide screening and characterization of the *GS* gene family has been performed in several plants following the release of high-quality reference genomes. It has been generally accepted that *GS*s play a large role in nitrogen metabolism. For example, with the help of whole genome sequencing data, *Populus trichocarpa* was the first plant species in which the complete *GS* family was observed to be duplicated (Castro-Rodríguez et al. 2011). Liu et al. (2018) identified six *GS* genes from *Gracilariopsis lemaneiformis* genome through transcriptome data and all of these genes were divided into three clusters, and found that GSII might have a key role in the process of nitrogen metabolism. Moreover, Czyż et al. (2020) studied the complex characterization of narrow-leafed lupin *GS* gene family with advanced genomic resources and pointed out that sub-functionalization and/or regulatory rewiring played an important role in shaping the primary metabolic pathways of the extant carbon and nitrogen in some lineages. However, very little information about *GS* from *Ta* and its relatives is available. Nowadays, the genomes of *Ta*, *Td*, *Aet*, and *Tu* have been better sequenced for further understanding of wheat genomics (IWGSC 2018; Avni et al. 2017; Luo et al. 2017; Ling et al. 2018).

A previous study identified 10 *GS* genes in wheat using heterologous complementation and cloning, which is the first cloning and study of *GS* genes in wheat (Bernard et al. 2008). In this study, total of 15, 9, 6, and 5 *GSs* were identified in *Ta*, *Td*, *Aet*, and *Tu*, respectively (Table 1). In previous studies, plant *GS* genes were organized in 4 groups, 3 of which code for cytosolic isoforms (GS1) and 1 codes for the chloroplastic isoform (GS2). Our results indicate that the family members are organized in 5 groups, with clade III, IV, and V belonging to GS1 and clade II belonging to GS2, in addition, 9 genes belong to clade I showing a high identity level to the *GS* genes in micro-organisms (Fig. 3). Rodríguez et al. (2011) also found that some *GS* genes clustered with archaebacteria. The hexaploid wheat (*Ta*, BBAADD) established from *Td* (BBAA) and *Aet* (DD) less than 10,000 years ago (Marcussen et al. 2014; Feldman et al. 1995). The tetraploid wheat (*Td*) was produced through domestication by the wild tetraploid wheat *T. turgidum* ssp. *dicoccoides* (BBAA), which was formed via allotetraploidization from *Tu* (AA) and *Aegilops speltoides* (BB) about 0.5 million years ago (Marcussen et al. 2014; Dvorak et al. 2005). For the number of *GSs* in each isoform, *Ta* wheat had nearly 1.5 times as many as *Td* wheat and 3 times as many as *Tu* wheat (Table 1) and the *GSs* located in each genome corresponds one by one (Fig. 2). Furthermore, the synteny analysis illustrated that most *TdGSs* and *AetGSs* had intergenomic homologous genes in *Ta*, while two of five *TuGSs* had homologous genes in *Td* and *Ta.* These results also supported the evolutionary relationship between diploid, tetraploid and hexaploid wheat.

Numerous studies have shown that GS can regulate nitrogen metabolism in plants and affect development and growth (Migge et al. 2000; Oliveira et al. 2002; Thomsen et al. 2014). Bernard et al. (2008) also suggested that wheat cytosolic isozymes (GS1) played a major role in assimilating ammonia during the critical phases of remobilisation of nitrogen to the grain. However, the involvement of GS in tolerance to abiotic stress has rarely been investigated. The present study focused on *TaGS* genes that could play crucial roles in abiotic stress tolerance. Combining the transcriptome expression of *TaGSs* genes, *Ta4D.GSe* was selected to further understand its characteristics and functions. For the localization analysis, Ta4D.GSe was localized in cytoplasm (Fig. 8), such a result exactly meets the characteristic of cytosolic GS1 isoenzyme. Transcriptome data showed that *Ta4D.GSe* was expressed in almost all organs, particularly in spikelets (Fig. S1). In addition, Ta4D.Gse-OE showed a higher germination rate under mannitol stress and drought rehydration survival rate when exposed to repeated drought treatments (Fig. S2; Fig. 9), which were associated with the function of *Ta4D.Gse* for osmoregulation and ROS scavenging. Under drought treatment, Ta4D.GSe-OE lines accumulated much more soluble sugars and free Pro (Fig. S3), which could regulate osmotic pressure and protect the integrity of cell membranes as osmoprotectants. Futhermore, the free Pro could also directly neutralize ROS and might scavenge ·OH (Sharma and Dieta 2006; Hayat et al. 2012; Signorelli et al. 2014). In addition, GS could also resist stress by protecting the antioxidant system (Reddy et al. 2015). Before drought stress, there were small but significant differences in the activities of SOD, POD and CAT between Ta4D.GSe-OE and WT; however, the enzyme activities differed greatly between Ta4D.GSe-OE and WT, after drought stress (Fig. S3), indicating that *Ta4D.Gse* could stabilize antioxidant system, thereby diminishing the impacts of ROS. Taken together, these results suggested that *Ta4D.GSe* may be involved in drought tolerance. However, the detailed correlation between *GSs* and drought tolerance remains to be further verified.

## Conclusions

In this study, 6 *AetGSs*, 15 *TaGSs*, 9 *TdGSs* and 5 *TuGSs* were identified and clustered into five lineages according to the phylogenetic tree. Particularly, according to the published sequence of micro-organisms nine novel *GSs* were found, and expressed them as *GSm1*. Then, their chromosome location, conserved motif, gene structure, and synteny were analyzed for understanding the gene family expansion and gene evolution. In addition, we used transcriptome data of Qingmai6 under abiotic stress conditions (drought and salinity) to identify the *TaGSs* expression profile, implying that *Ta4A.GSe*, *Ta4B.GSe*, and *Ta4D.GSe* might be involved in the response abiotic stress. Because of its high expression level, *Ta4D.GSe* was selected as representative gene for further functional investigation. The subcellular location of Ta4D.GSe to the cytoplasm was detected using confocal microscopy. Furthermore, its functions involved in abiotic stress were identified by inducing its overexpression in *At.* The results showed that, *Ta4D.GSe* plays an important role in conferring drought tolerance by improving the scavenging of ROS and the osmotic adjustment ability in *Arabidopsis*. Taken together, these findings provide insight into the potential functional roles of the *TaGSs* genes in abiotic stress tolerance.

## Supplementary Information

Below is the link to the electronic supplementary material.

Supplementary file1 (XLSX 26 kb)

Supplementary file2 (TIF 1354 kb) **Fig. S1** Expression pattern of *Ta4D.GSe* in different tissues. **A** Expression levels of *Ta4D.GSe* in seedling stage. **B** Expression levels of *Ta4D.GSe* in maturation stage. The relative expression levels in each tissue were calculated by setting the expression value of *Ta4D.GSe* in root as 1. The relative expression values were calculated through the 2^−ΔΔCq^ approach

Supplementary file3 (TIF 12404 kb) **Fig. S2** Overexpression of *Ta4D.GSe* improves the relative germination rate of *Arabidopsis*. **A** Germination of *Arabidopsis* treated with 150 mM mannitol. **B**–**D** The relative germination rate of *Arabidopsis* treated with mannitol at 50, 100, 150 mM

Supplementary file4 (TIF 5092 kb) **Fig. S3** The differences of physiological indexes before and after treatment of drought in *Arabidopsis*. *WT* empty vector lines, *OE Ta4D.GSe* overexpression transgenic lines. **A** Identification of the glutamine synthetase (GS) activity in WT and OE before and after drought treatment. **B**–**D** Activity of SOD (**B**), POD (**C**), CAT (**D**) in *Arabidopsis*. **E** and **F** Changes of soluble sugars and proline content of *Arabidopsis* under drought treatment

## Data Availability

The datasets supporting the conclusions of the present study are included within this article (and its additional files). The authors are pleased to share any raw data upon request. Not applicable.
