# Supplementary figures and images for: Identification of the glutamine synthetase (GS) gene family in four wheat species and functional analysis of Ta4D.GSe in Arabidopsis thaliana
Source: Plant Mol Biol. 2022 Jun 18;110(1-2):93–106. doi: 10.1007/s11103-022-01287-4 (PMC9468116; doi:10.1007/s11103-022-01287-4)

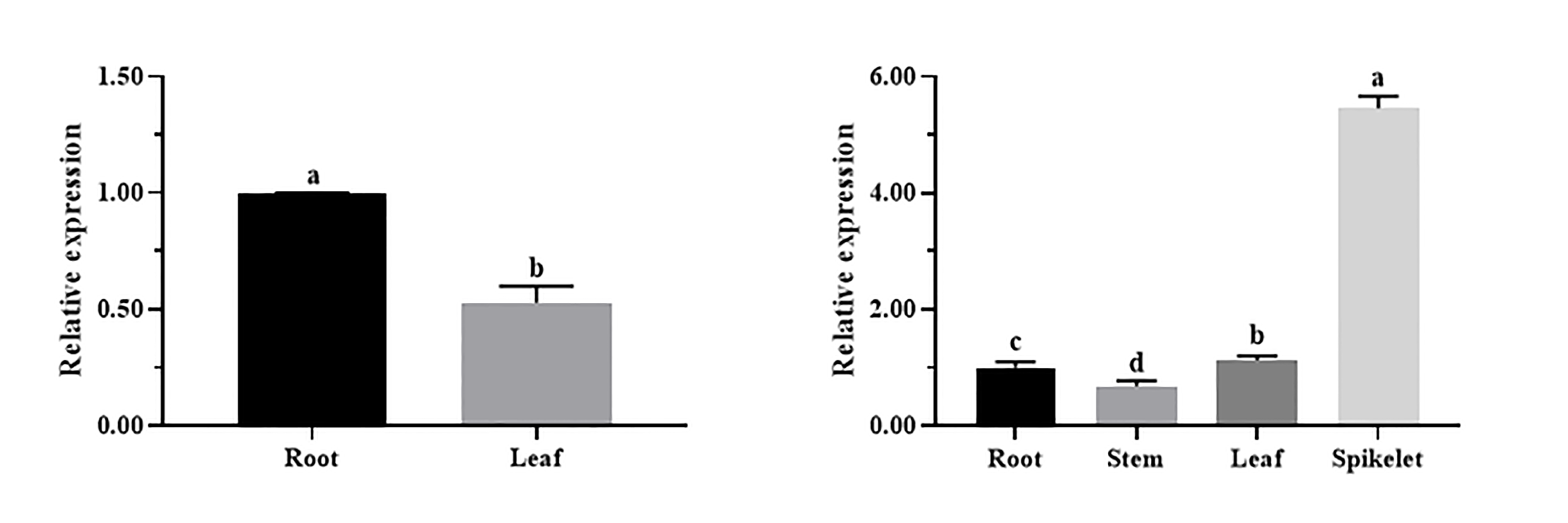

Supplement: Supplementary file 2 — Supplementary file2 (TIF 1354 kb) Fig. S1 Expression pattern of Ta4D.GSe in different tissues. A Expression levels of Ta4D.GSe in seedling stage. B Expression levels of Ta4D.GSe in maturation stage. The relative expression levels in each tissue were calculated by setting the expression value of Ta4D.GSe in root as 1. The relative expression values were calculated through the 2−ΔΔCq approach [file 11103_2022_1287_MOESM2_ESM.tif]

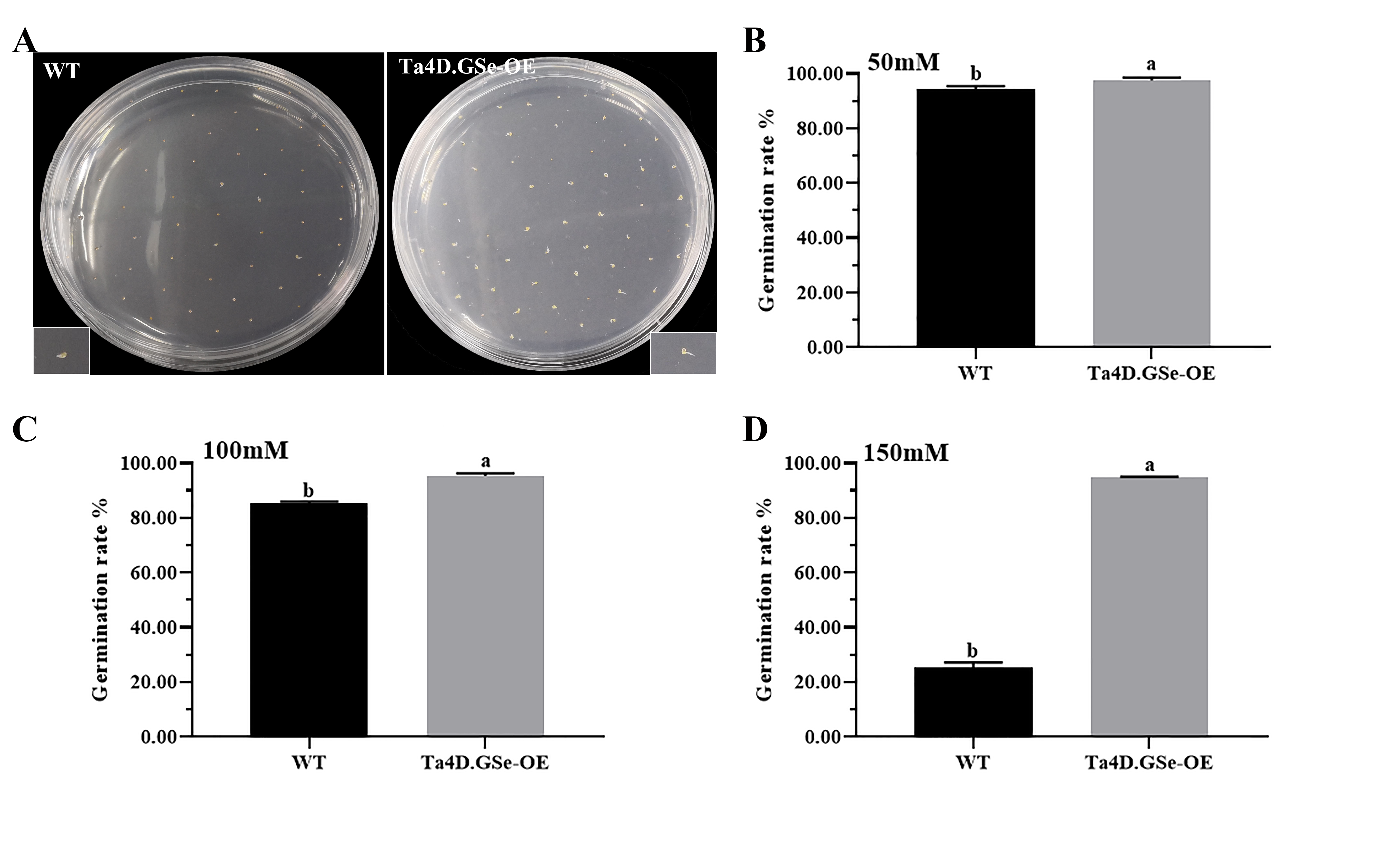

Supplement: Supplementary file 3 — Supplementary file3 (TIF 12404 kb) Fig. S2 Overexpression of Ta4D.GSe improves the relative germination rate of Arabidopsis. A Germination of Arabidopsis treated with 150 mM mannitol. B–D The relative germination rate of Arabidopsis treated with mannitol at 50, 100, 150 mM [file 11103_2022_1287_MOESM3_ESM.tif]

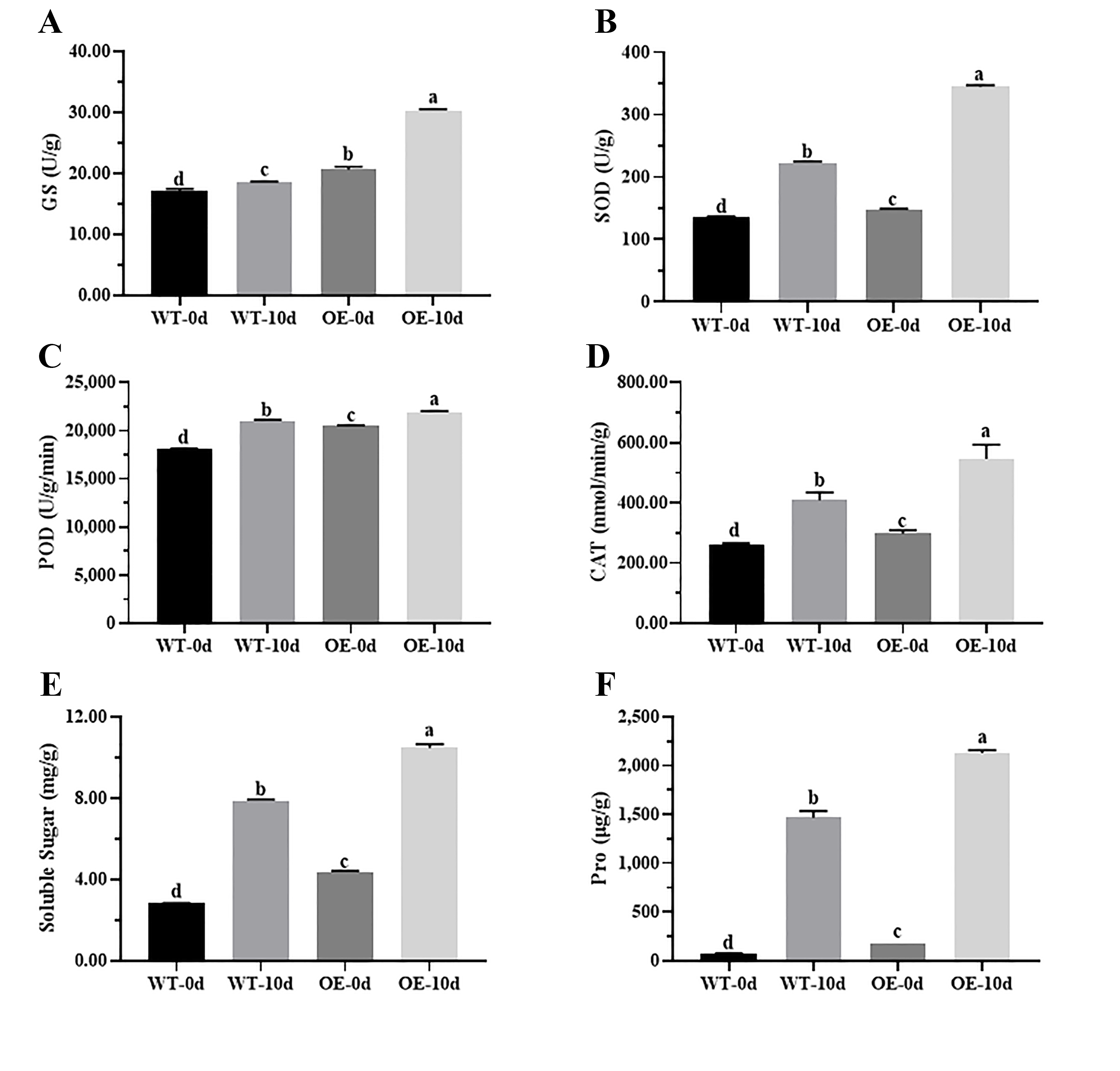

Supplement: Supplementary file 4 — Supplementary file4 (TIF 5092 kb) Fig. S3 The differences of physiological indexes before and after treatment of drought in Arabidopsis. WT empty vector lines, OE Ta4D.GSe overexpression transgenic lines. A Identification of the glutamine synthetase (GS) activity in WT and OE before and after drought treatment. B–D Activity of SOD (B), POD (C), CAT (D) in Arabidopsis. E and F Changes of soluble sugars and proline content of Arabidopsis under drought treatment [file 11103_2022_1287_MOESM4_ESM.tif]
